# Chat (Catha edulis): a socio economic crop in Harar Region, Eastern Ethiopia

**DOI:** 10.1186/2193-1801-3-579

**Published:** 2014-10-03

**Authors:** Laxman S Kandari, Hiranmai R Yadav, Ashok K Thakur, Tripti Kandari

**Affiliations:** School of Natural Resource Management and Environmental Sciences, College of Agriculture and Environmental Sciences, Haramaya University, P.O. Box # 337, Dire Dawa, Ethiopia; School of Plant Sciences, College of Agricultural and Environmental Sciences, Haramaya University, P.O. Box # 138, Dire Dawa, Ethiopia; Wildlife Institute of India, P.O. Box No. 18, Chandrabani, Dehradun, 248 001 Uttarakhand India

**Keywords:** Agriculture, *Catha edulis*, Oromiya, Socio-economic impact

## Abstract

Chat (*Catha edulis*) is an important perennial crop and its leaves are chewed for a stimulating effect. It is widely cultivated in the Ethiopian highlands of Oromia region and is figured as Ethiopia’s second largest foreign exchange earner. Its cultivation accounts for about 70% of farmer’s income in the study area. The common effect of its consumption leads to insomnia, a condition that the users sometimes try to overcome with sedatives or alcohol. The present study is an attempt to survey and assess the impact of crop on the community. It has been observed to implicate health problems, reduces savings and nutritional standards of the family members. The chat yields in the area ranges from 1500–1800 kg/ha through monoculture. During the study, the average monthly income of the family practicing chat cultivation was from Birr 8, 533.00 to 13, 166.00 kg/ha per year in Baate and Genede cultivating areas. When the average cost per/ha was rupees 60/kg. The present study shows that during the recent past, leaf consumption has increased significantly. Chat growers are not only producers but also traders and consumers. Its consumption has become a widespread habit from secondary schools. Highest number of consumers was found to be among drivers followed by students and shopkeepers. The consumption of the plant is not considered a taboo but on contrary a status symbol in the region. It has no legal or moral implications and is considered as a part of custom and habit of local people. High value cash crop like vegetables and orchard fruits needs to be used as a replacement for chat which could be a regular source of income to farmers. Alternative sources of income for farmers needs to be scientifically worked out and proposed keeping in view the proportion of agricultural land reserved under chat cultivation and to increase the production of food grains being produced.

## Introduction

**Figure 1:**
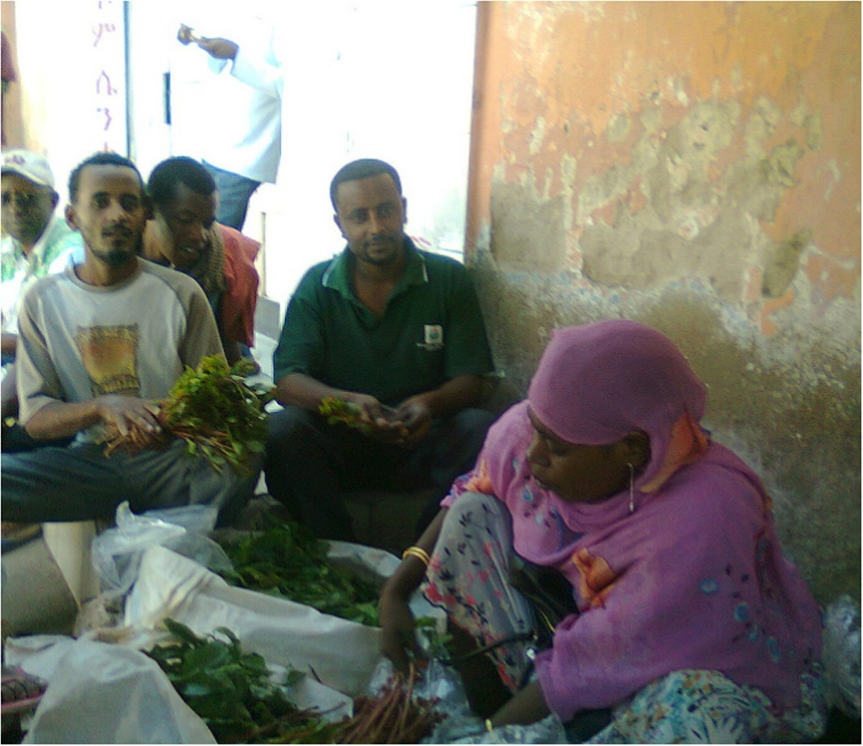
**Woman chat seller selling chat to local people.**

Chat (*Catha edulis* Forsk), or Quat belonging to family Celastraceae is considered an evergreen plant, cultivated for the production of leaves having sympathomimetic actions which are used commonly for gradual chewing (Cox and Rampees 2003). The bole is straight and cylinder. The bark is whitish and crown is small. Flowers are small and white in colour with five petals and five stamens in axillary cymes 1–8 cm long. The calyx is five lobed; the capsule is oblong, woody, pendulous about 1 cm long with 3 valves. This plant is called by different names in different countries: ‘chat’ in Ethiopia, ‘qat’ in Yemen, ‘mirra’ in Kenya and ‘qaad’ or ‘jaad’ in Somalia. In most of the literature, it is largely known and called as chat (Belwal and Teshome 2011). In Ethiopia, this is grown extensively in the middle altitudes between 1500 and 2100 meters above sea level (masl), and performs better on well-drained soil under diverse climatic conditions. It can tolerate drought conditions for several months. The crop can be harvested around the year, thereby becoming a source of continuous revenue for the farmer. The economically important parts of the plant are its young leaves and tender twigs, which are chewed for their stimulating effect. It is not uncommon to come across many farm ladies selling their plants to willing buyers in the local market throughout the day (Figure 1). Chat production and consumption occupy a major area in eastern Africa, South-west Arabia, and Madagascar (Pantelis et al. 1989). Its consumption can alleviate fatigue, promote excitation, increase confidence and suppress sleep and hunger (Al-kamel 2001).

**Figure 2:**
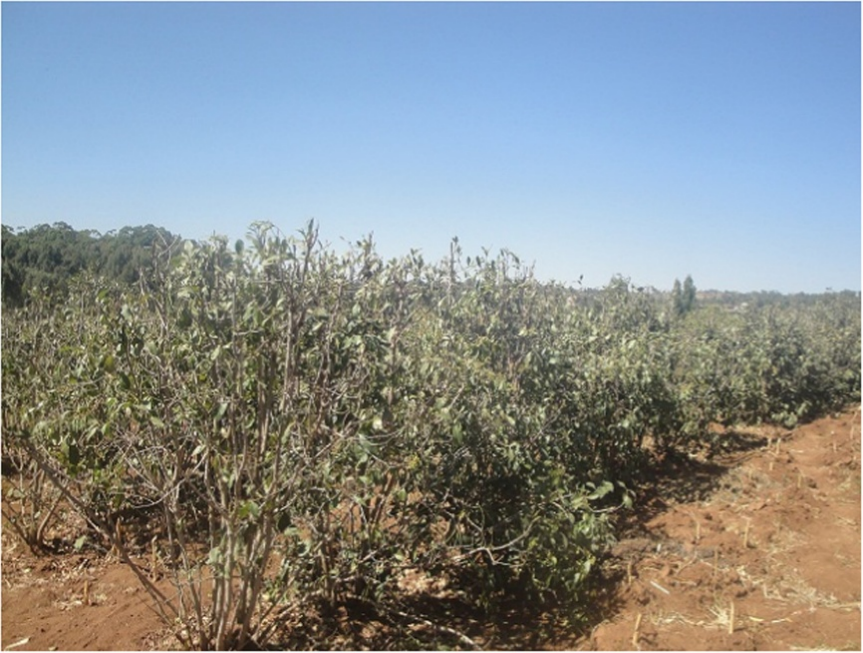
**Intercropping of chat with Sorghum.**

Ethiopia is the world’s largest producer of chat which has recently become the fastest growing export commodity. The history of domestication and introduction of this crop in Ethiopia is not known. According to the folklore, it was first introduced in Harar from where it spread to rest of the country (Getahun and Krikorian 1973). Harar is located in eastern Ethiopia. The main inhabitants of this region are Oromia tribal communities. About a third of the production is exported to neighboring countries like Djibouti and Somalia. However, it is largely produced, marketed and consumed within the country. Harar is a business centre where the plant is grown at a wide range of altitudes, soil types and climatic conditions (Alles et al. 1961; Beguinot 1939; Beitter 1900; Brooke 1960). Cultivation of this crop has helped to a good extent to reduce poverty from the region. The price of chat fluctuates seasonally according to the quality and quantity supplied. The price is higher in the dry periods due to short supply but decreases during the rainy season (from April to September), when the supply is more. Currently, chat crop is grown in about 94,330 ha of land nationwide and accounts for about one third of the area under coffee (Anonymous 2000). The bulk of the chat produced in Harar region is of good quality and is in great demand in both domestic as well as in export markets. It is now a major source of foreign exchange and occupies second place in export after coffee. In 1998–99, the crop accounted for 13.4% of Ethiopia’s export earnings and was the country’s second largest export items that year (CSA 2000). Consumption of chat leaves is common in Middle-East Asian countries like Yemen, Saudi Arabia and East African countries like, Kenya, Somalia, Djibouti, Uganda and Tanzania. Normally chat saplings are first planted around the homestead and later expanded to rest of the farmland. Cultivation of chat occupies 55% of agricultural land and is largely intercropped either with maize or with sorghum (Figure 2).

Many users report that feeling of happiness associated with better thinking capacity. Currently, majority of the farmers are involved in chat cultivation in this region. It is widely enjoyed by both male and females and is commonly used for social recreation and family ceremonies. Occupational groups such as motor vehicle and truck drivers chew plant leaves regularly during long distance driving and admit that it keeps them alert and awake. A significant number of students have also been observed to consume the leaves to remain alert especially during examination periods. There is also a specific usage of this plant by the special sections of the community like craftsmen and workers in small scale industries use it to reduce physical fatigue and traditional healers to heal ailments. The plant is interpreted as a stimulant of physical and psychological functions. Its psychic influence depends on its active ingredients that have a stimulating and cuphoric effect. The plant is chewed for a stimulating effect that is similar to that of amphetamine. Cathinone, found in fresh leaves is listed as a schedule I drug in the United States in the same group as heroin and cocaine (Brooks 1997). During maturation and decomposition of the plant, cathinone is converted to cathine, a Schedule IV drug (legal). In fact, for a variety of reasons, the habit of chat consumption is deeply rooted in social and cultural traditions of Yemen, Ethiopia, Somalia, and Djibouti (Getachew 1996; Bali 1997; Klingele 1998). The present study is an attempt to survey and assess the impact of crop on the community.

## Materials and methods

### Study area

**Figure 3:**
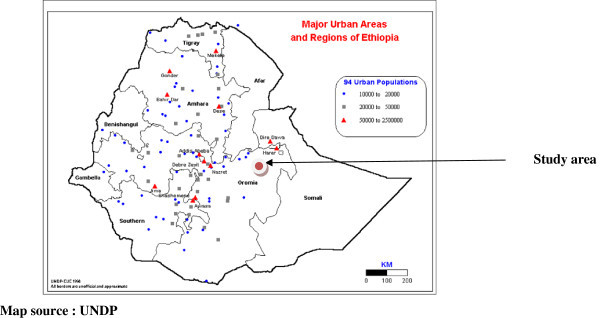
**Map of the study site.**

The present study area encompasses the Haramaya town and adjacent areas in eastern-Ethiopia (Figure 3). The information and findings presented are primarily based on the interviews through a prepared questionnaires held with the traders and clients in the Baate and Gendaje areas of Harar and other consumers belonging to all categories of people i.e., local people, drivers, shopkeepers and students. The trading of chat is highly pronounced in the Harar region. Although people are subjected to modern facilities, the multi-ethnic composition of the population still clings to traditional beliefs and practices that are deeply rooted in their mind and culture.

### Selection of informants

A reconnaissance survey was undertaken during 2011–2012 in the market and nearby adjoining area of Harar region i.e., Baate and Gendaje and where the plant is commonly sold. During the survey, attempts were made to collect all possible information regarding the traditional and ethnobotanical use of plant in the region; mode of usage and part of plant used. A semi-structured questionnaire survey with focused group discussion (FGD) were conducted with different groups of people i.e., among all categories of people. Most of the local people identified during the field survey were in the age category of 20–40 and > 50 years who were habitual and willing consumers. All of them were familiar with the use of the plant for cultivation and its subsequent consumption pattern.

## Results and discussion

### Botany and phenology of the *Catha edulis*plant

**Figure 4:**
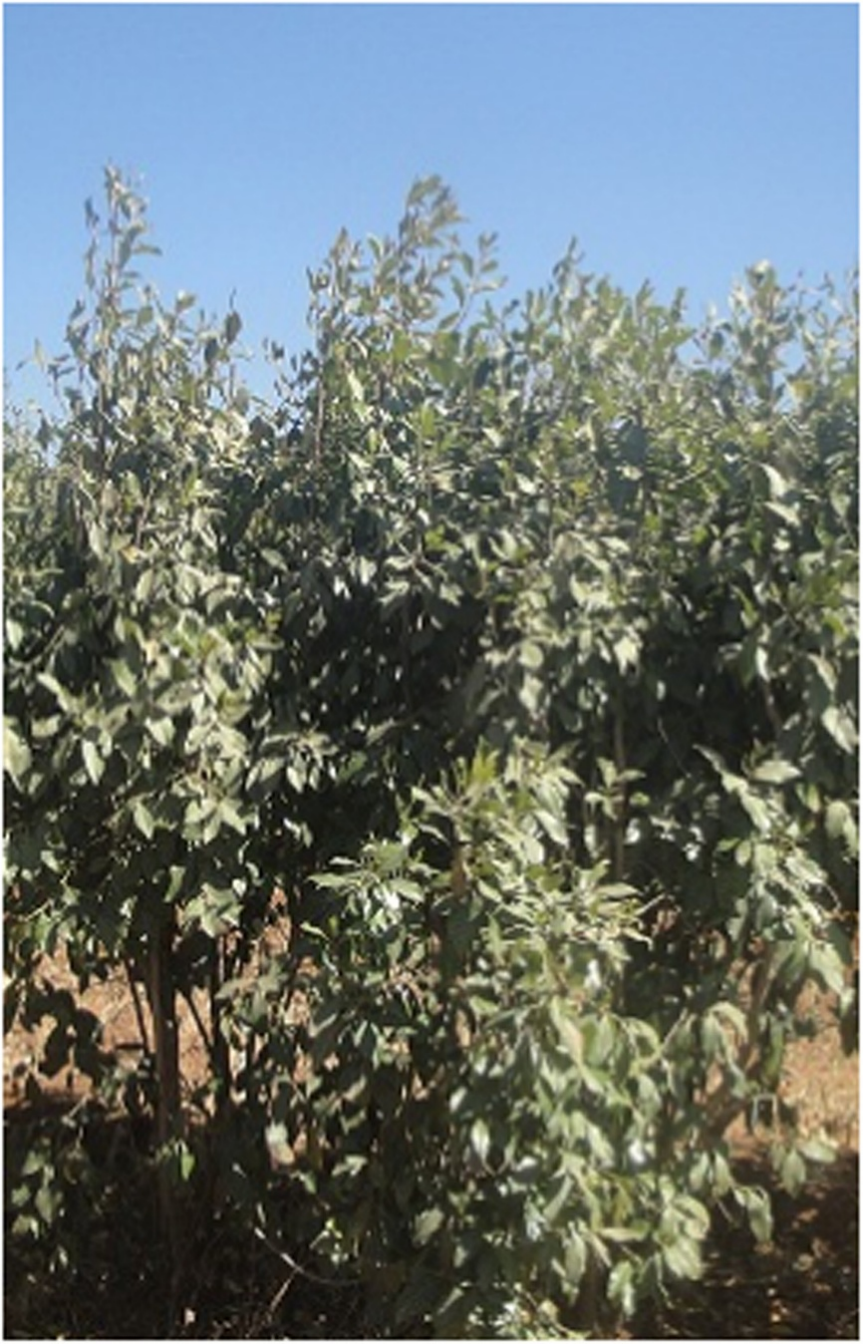
**A full mature chat plant.**

*Catha edulis* is a flowering evergreen large shrub of Celastraceae family. The plant usually grows at high altitude areas (1500–2500 m asl) in Ethiopia, Kenya, Yemen, South Africa and Madagascar. The plant reaches up to 25 m in height when grown naturally (Klingele 1998), but it is kept under manageable height when grown as a cash crop to facilitate convenient height for harvest and good economic return (Figure 4).

### Socio-economic effect and expansion of chat production

**Figure 5:**
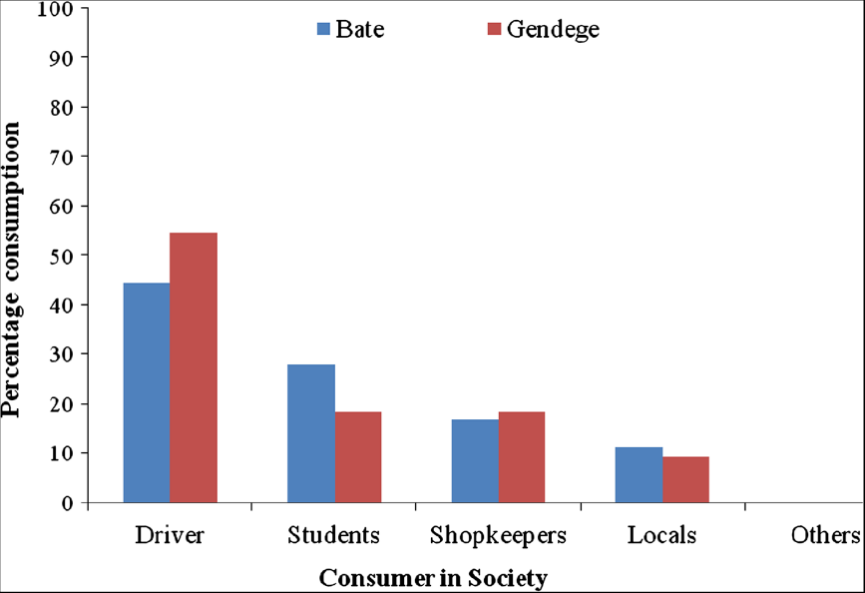
**Consumption of chat by different sector of society.**

**Figure 6:**
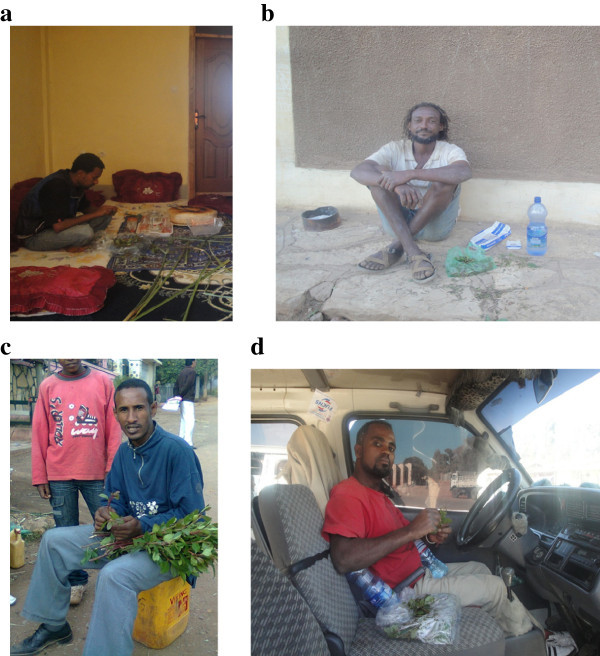
**Local people enjoying chat. (a)** Enjoying chat in home. **(b)** A labour enjoying chat in free working hours.

It is observed that chat growers are not only producers but also traders and consumers. Its consumption has become a widespread habit from secondary schools. Highest numbers of consumers were found to be among drivers followed by students and shopkeepers (Figure 5). The leaves intake in general, negatively affects the working ability and capacity of the users. In fact, its consumers have negative health effect with positive income effect. As, such, policy measures required to control its cultivation will need to address both supply and demand issues in relation to its negative impact on the society. Chewing of leaves has been practiced for social and psychological reasons for many centuries and its use has gradually expanded worldwide. Both socioeconomic and agro-ecological reasons have contributed for its expansion and production thereby increasing market opportunities (Figure 6). Production of the plant is mainly located close to the road network and on farms with irrigation facilities. It has assumed the status of cash crop with an assured income. The profitability of its production is, therefore, considered as the primary reason by 75% of the farmers for expansion of cultivation in the area. The income generation from intercropping with maize is another added advantage of high returns as compared to mono-cropping of agriculture. Income from maize mono-cropping is less as compared to intercropping system, which provides 3 times higher return. Due to assured and higher returns from cultivation of the plant as well as low income from other crops or mono-crops, many farmers (having small landholdings) have expanded its cultivation in their fields. The other factors contributing to the expansion of this crop includes low risk and low labor intensive compared to cereal crops. Ownership of chat area under cultivation is considered as one of the important indicators of prosperity when a man proposes marriage to a woman in this region. The production of this crop has expanded at the expense of important rainfed cereal crops like maize, sorghum, coffee etc. and serves as a good substitute, as it is less vulnerable to drought. The cultivation of cereals is expensive, as it requires fertilizers and irrigation. The crop being perennial, farmers in the area considered this as a way to ensure land entitlement because annual cropland is more affected by land redistribution than land under perennial crops.

Agricultural labours also show more interest to work in chat cultivated fields as its cultivation offers wage or bonus to the workers. Credit is also available more easily for chat growers as the income is more or less assured. It has become the major source of income in the region and constitutes approximately 70% of a farmer’s income. It was found that nearly 3.5–4.28 numbers of family members out of 5 were involved in cultivation of this plant in the area Gendaje and Baate (Table 1). The chat yields in the area ranges from 1500–1800 kg/ha through monoculture. During the study, the average monthly income of the family practicing chat cultivation was from Birr 8, 533.00 to 13, 166.00 kg/ha per year in Baate and Genede cultivating areas. When the average cost per/ha was rupees 60/kg. The present study shows that during the recent past, leaf consumption has increased significantly. Its cultivation continuous to play an important role in the social life of people in most parts of Ethiopia particularly in celebrations, marriages, business and political meetings (Figure 5). A survey of the Harar region revealed that, the use of plant leaves has become popular among all segments of the population and different age groups (Selassie and Gebre 1996). Its use was previously limited to its producing areas, which has now spread to all parts of the country. It is now air freighted and available in Europe, Canada and the United States, following the migration routes of immigrants from east Africa and southern Arabia.

**Table 1:** **Socio-economic dependency of people on chat**

S. No.	Parameters	Villages	
		Genede	Baate
1.	Number of families members involved in chat business	3.5 ± 0.88	4.28 ± 0.821
2.	Number of families have their own land	33.3 ± 0.166	21.42 ± 0.10
3.	Working hrs per day	14.8 ± 0.77	14.4 ± 0.77
4.	Monthly income	131, 66.00 ± 5296	8,533.00 ± 890.21
5.	Price of one bunch of chat	58 ± 9.27	41.32 ± 6.412

±Standard error.

Specific preference for cultivation of this plant has resulted in very glaring agriculture scenario in the area

1. Provision of cash crop which provides assured income to the farmers throughout the year at regular intervals.
2. Increase in goat population which could be raised with the little inputs that brings in revenue through meat sales during festive periods.
3. Raising employment opportunities through domestic and international trade along with increased transportation.
4. Easy availability of labour.
5. Negligible need for irregular and vital inputs.
6. Easy credit availability.
7. It enjoys a status of important foreign exchange earner.
8. The crop assured elevated social status for the producers.

### Impact and response of society and consequences

Development of withdrawal symptoms, comprising heavy and sinking sensatation following habit of prolonged consumption of chat seems to surfacing among consumers. The frequency of lethargy, mild depression, slight trembling and recurrent bad dreams prompt them to have second thoughts about its consumption. A serious consideration is that, its use may endanger health, thereby resulting in anorexia leading to malnutrition with subsequent susceptibility to infectious diseases. As a result of area expansion under this plant, the farmers need to buy cereals now to meet their food requirements and thus its production has resulted in problem of food insecurity in the region. The farmers’ personal production of food crops may last for 6–7 months only. Only about 51% of the households have enough food throughout the year. Chat consumption negatively affects the working capacity of people because they tend to be slow in work, show lethargy, less number of working hours, take frequent rests, spend time chewing the leaves, and are generally more careless and found loitering about aimlessly in the market. Its consumption has become a common habit among the population above 13 years of age including the secondary school students. Consumers spend a high portion of their income to purchase these leaves which leads to a serious under nourishment of the poor people leading to social consequences. The number of goat population has increased in the current farming system due to chat foliage availability and less dependence on crop residues for feed along with different fodder requirements. The labour requirement for chat cultivation is lower than cereal mono-cropping with total volume of employment rising due to ancillary post harvest activities, trade and transportation. In Somalia, it is reported that the plant consumption is promoting different types of criminal activities (Elmi et al. 1987).

### Its increased popularity however has brought in important social problems in its wake

1. Health problem like mild depression and trembling
2. Malnutrition and susceptibility to infectious diseases
3. Low food grain production resulting in increased dependence on market for food grains.
4. Affected chewing capacity of people accompanied with lethargy
5. Affected school children resulting in school dropouts
6. Increase in criminal activities
7. Decrease in area of cultivation under cereal crops with subsequent drop in production of food grains.
8. Due to fall in area under fodder crops the livestock population along with milk production has been on a downswing

The only positive effect of chat production is higher income generation for farmers, more employment generation to the rural people engaged in chat transportation and trade related activities.

### Chat expansion on the farming system

It has been observed that production of this crop in the study area has expanded to almost 55% of the cultivated area in Harar which includes intercropping with maize and (to some extent with) sorghum. In the intercropping category, this plant has been found to have the largest share of land resulting in decreases in yield of maize and total cereal production in the area. The expansion of area under chat production has also changed the composition of livestock system in the region due to decreased fodder availability and less interest in livestock raising. The need for drought animals like oxen has reduced in the ongoing pattern of chat-based cultivation system. Further, fodder availability has been reduced because chat occupies the larger share of the land coupled with low animal population. Fodder species like *Erythrina abyssinica*, *Sesbania sesban* and *Leucaena leucocephala* are completely replaced or removed. Shifts in the cropping pattern also resulted in decreased number of cows with decreasing milch cattle and the milk availability in the market is currently on the downswing.

### Intercropping

In the area surveyed rainfed farming is undertaken and farmers need food crop for their annual domestic consumption needs for which Sorghum and sweet potato is cultivated. Maize is sometimes also cultivated instead of Sorghum depending upon seed availability. Sorghum, corn and sweet potatoes are generally accepted for intercropping with chat which is a cash crop. This is possible as these plants are so spaced that they leave much area in between the rows (Figure 2). The preparation of the seedbed required for intercropping with Sorghum provides higher and additional benefits. Thus, the intercropping is practiced to improve the economy of the farmers. On the other, chat leaves could be collected and marketed at regular intervals unlike most agricultural crops that are seasonal which is a welcome source of the otherwise poor cultivation. Vegetables crops are not preferred in this region and are cultivated where water is available.

### Biochemical constituent

The active ingredient of chat responsible for its psycho stimulant effect is an alkaloid chemical known as cathionine, which is structurally and chemically similar to d-amphetamine and cathine a milder form of cathionine. Cathionine is a highly potent stimulant, which produces sympathomimetic and central nervous system stimulation analogous to the effect of amphetamine. Fresh leaves contain both ingredients: those left unrefrigerated beyond 48 hours would contain only cathine, and it supports user’s preference for fresh leaves. The plant loses its potency after 48 hours. The results of various *in-vivo* and *in-vitro* experiments indicated that the substance could be considered as a natural amphetamine. The active constituent, cathinone, which causes sympatno-mimetic effects and symptoms such as euphoria and hyperactivity, is released with following consumption of leaves.

Cathinone has analogous mechanisms of action with pharmacological properties that are reminiscent of those induced by amphetamine, i.e. anorexia as well as hypermotility (Valterio and Kalix 1982). In fact, it is now being referred to as a “natural amphetamine” and its effects in animals correspond to those observed in human consumers (Alem et al. 1999). The World Health Organization (WHO) has included and reported cathinone in its list of controlled drugs and equivalent to amphetamine abuse (WHO 1980; Kalix 1981). The plant leaves are considered rich in ascorbic acid, which minimizes the undesirable side-effects of its chewing. By modulating catecholaminergic activity or transmission of dopamine, ascorbic acid acts as an antidote to the effects of amphetamine (Broody 2002; Gulley and Rebec 1999). It is assumed that there is an adequate quantity of ascorbic acid in the leaves, which is released from the leaf matrix by mastication and becomes bio-available. Ascorbic acid, a well-known antidote against amphetamine and amphetamine-like substances, was reported to reach as much as 325 mg/100 g leaf of chat (Krikorian and Getahun 1973).

In light of World Health Organization (WHO) recommendation, the problems associated with chat-chewing for the moment should be considered in a manner similar to amphetamine abuse. Hence, it is the psychotropic and mind-altering drug type whose use could possibly constitute risk behavior in the amplification of the HIV/AIDS epidemic in countries like Ethiopia, where the habit is widespread (Abebe et al. 2005).

Keeping in view the adverse side effect of consuming chat at fall out on the health of younger generation of the region, it is imperative that crucial step be undertaken for production and consumption of this plant.

### Recommendations

Since, impediments in its cultivation would affect the economy of the area, the following point need to be considered for implementation.

1. Legislation need to be enforced for confining its cultivation to a particular region.
2. Government machinery needs to be in place for collection of harvest at market support price and for meeting exports.
3. Proper guidelines should be placed for its consumption. Younger generation should be prohibited from consuming it
4. Providing education for the students to stay away from its consumption by highlighting its side effects.
5. Establishing government laboratories for analysing the plant and giving the task of enforcing ban on its consumption by younger generations.
6. Establishing chat free areas in the country.
7. Creating revenue earning demonstration areas of vegetable and flower cultivation to wean away the farmers from the chat.

In Ethiopia, farmers have few alternative sources of income with similar profitability. Control on chat will therefore have to address both supply and demand. Alternative sources of income for farmers need to be designed and thoroughly investigated while providing other appropriate options. Education and awareness programs about consumption and its negative consequences may reduce the demand for chat.

## Conclusion

From the current study, it is concluded that, chat production is expanding rapidly around Harar region, particularly at the expense of important cereal crops. The economic benefits and easy cultivation of the crop is a strong compelling reason for its expansion and increasing the income nearly 70% of farmers of the region. The crop being perennial, reduces the frequency of ploughing i.e., once in three months or yearly. Therefore, farmers are interested to cultivate it instead of food crops and to have more goats instead of oxen. Goats need less fodder and are more income generating. Chat producers become its perpetual users and its consumption is widespread among locals (both male and female) and even students. The expansion of this crop production in Ethiopia may bring considerable social and economic risks in the longer run as earnings totally depend as the plant is banned in many other countries. The consumption of this crop generates health problem and reduces the productivity in terms of work efficiency and number of working hours. The increased use of the plants needs to be monitored and controlled particularly among the younger generation who are being otherwise talked into imaginary well being. It is a evident from this study that the plant is very popular among all age group of people approached. The consumption is not common among people beyond 60 years of age. It has been supplementing the income of farmers in the absence of other cash crops. The crop now enjoys a well developed flourishing market which is instrumental in reducing poverty in the area.

### Consent

Written informed consent was obtained for the publication of this report and any accompanying images.
